# Case of Extirpation of an Ovarian Cyst

**Published:** 1844-04-06

**Authors:** Bransby B. Cooper

**Affiliations:** Surgeon to Guy’s Hospital


*Case of Extirpation of an Ovarian Cyst.* By Bransby B. Cooper, F. R. S., Surgeon to Guy's Hospital.

The patient was aged 32, and married for four years, without having children. She had suffered at different periods from dysmenorrhœa, and leucorrhœa. Five years before her admissisn her abdomen became greatly enlarged, and, having consulted the author, he considered the case to be one of ovarian tumour, and proposed to draw off the fluid from the cyst, and then remove the cyst by a small opening in the abdomen. But this plan was not carried into effect. She was afterwards tapped on two different occasions, when about three gallons of straw-coloured fluid were discharged each time. When she applied at the hospital, earnestly soliciting to have the operation of removing the tumour performed, her abdomen measured about three feet and a half in circumference. Mr. Cooper resolved to perform the major operation. An incision was, therefore, made through the abdominal parietes, from the ensiform cartilage to the pubis. A few adhesions to the tumour were met with near the point where the trocar had entered in tapping; these having been divided, the cyst was dislodged and brought through the wound, when the pedicle connected with the right ovary came into view. A double ligature was passed through the pedicle, and both threads having been tied, the pedicle was divided between the cyst and the ligatures. The left ovary was examined, and found to be healthy. The wound was then brought together by sutures and strapping, and a roller applied round the abdomen. There were some attempts to vomit, but otherwise the patient bore the operation well. A minute record was given of the daily condition of the patient subsequent to the operation; but it is sufficient to say that distinct symptoms of peritonitis soon appeared, and the patient died on the seventh day, without any indication of the system rallying.

On the post-mortem examination, lymph was found effused extensively over the abdominal parietes and viscera, uniting them in many places together. A small piece of omentum was enclosed in the ligature applied to the pedicle. The uterus was large, tumid, and of a dark colour; and a soft fungous tubercle, pronounced, after microscopical examination, to be malignant in its structure, was found on its fundus. The cyst which was removed was of an oval form, and at its superior and anterior part there was a collection of compound cells. Its weight was thirty-two pounds.

The author concluded by making some general observations on the operation. He drew attention to the peculiar kind of inflammation which followed, and was inclined to think that it was excited more by the presence of the ligature applied to the pedicle, and left in the wound, than to the extensive incision. He also noticed the fact of a tumour of fungoid structure having been found growing from the substance of the uterus, and thought that this was interesting as illustrating the views of those pathologists who believed ovarian tumours, consisting of cysts to be malignant in their nature, and as bearing on the question of the propriety of removing such tumours by operation.—*Lon. Med. Gaz.*, Jan. 19, 1824.

